# Serum tumor necrosis factor‐α, interleukin‐1β, interleukin‐6, and interleukin‐17 relate to anxiety and depression risks to some extent in non‐small cell lung cancer survivor

**DOI:** 10.1111/crj.13457

**Published:** 2021-11-09

**Authors:** Meifang Liu, Yao Li, Xuesong Liu

**Affiliations:** ^1^ Department of Respiratory and Critical Care Medicine The Second Affiliated Hospital of Harbin Medical University Harbin China; ^2^ Department of Hematology The Second Affiliated Hospital of Harbin Medical University Harbin China; ^3^ Ministry of Nursing The Second Affiliated Hospital of Harbin Medical University Harbin China

**Keywords:** anxiety, depression, interleukin‐1β, non‐small cell lung cancer, tumor necrosis factor‐α

## Abstract

**Introduction:**

Inflammatory cytokines are proposed as modulators for the pathogenesis of anxiety and depression (anxiety/depression), and anxiety/depression are frequently existed in non‐small cell lung cancer (NSCLC) survivors. However, no published study has explored the association of inflammation cytokines with anxiety/depression in NSCLC survivors.

**Objectives:**

We aimed to evaluate serum tumor necrosis factor‐α (TNF‐α), interleukin‐1 beta (IL‐1β), interleukin‐6 (IL‐6), interleukin‐17 (IL‐17) levels, and their correlations with anxiety/depression in NSCLC survivors.

**Methods:**

Totally, 217 NSCLC survivors and 200 controls were recruited. Then, inflammatory cytokines in serum samples were detected by enzyme‐linked immunosorbent assay (ELISA). Besides, their anxiety/depression status was assessed by Hospital Anxiety and Depression Scale (HADS).

**Results:**

HADS‐anxiety score, anxiety rate, anxiety severity, HADS‐depression score, depression rate, and depression severity were all increased in NSCLC survivors compared with controls (all *P* < 0.001). Regarding inflammatory cytokines, TNF‐α, IL‐1β, and IL‐17 levels were higher (all *P* < 0.01), while IL‐6 (*P* = 0.105) level was of no difference in NSCLC survivors compared with controls. Furthermore, TNF‐α, IL‐1β, IL‐6, and IL‐17 were all positively associated with HADS‐A score (all *P* < 0.05), anxiety occurrence (all *P* < 0.05), HADS‐D score (all *P* < 0.05), and depression occurrence (all *P* < 0.05) in NSCLC survivors, while the correlation‐coefficients were weak. Additionally, multivariate logistic regression analyses disclosed that TNF‐α (both *P* < 0.05) and IL‐1β (both *P* < 0.001) were independently correlated with increased anxiety and depression risks in NSCLC survivors.

**Conclusion:**

Serum TNF‐α, IL‐1β, IL‐6, and IL‐17 are related to increased anxiety and depression risks to some extent in NSCLC survivors.

## 1 INTRODUCTION

Lung cancer represents as a leading cause of cancer‐related deaths worldwide, and a majority of lung cancer cases (approximately 85%) are non‐small cell lung cancer(NSCLC).^1^ Surgery is the primary curative treatment for surgical NSCLC patients, which provides a 5‐year survival rate of approximately 60%.^2^, ^3^ Meanwhile, owning to exacerbated symptoms (such as cough, wheeze, and dyspnea), declined physical functions, lifestyle changes, struggling to accept new treatment, and feeling of isolation after surgery, NSCLC survivors frequently experience mental discomfort that manifests as anxiety (ranging from 34.1% to 49.6%) and depression (ranging from 32.9% to 46.1%).^2^, ^4^, ^5^, ^6^, ^7^, ^8^ Furthermore, both anxiety and depression are associated with negative outcomes, such as reduced adherence to treatment, exacerbations of medical symptoms, declined life quality, and increased mortality in NSCLC survivors.^4^, ^9^ Therefore, the early identification and proper management of anxiety and depression are essential for the prognostic improvement in NSCLC survivors.

Inflammatory cytokines (especially tumor necrosis factor‐α [TNF‐α], interleukin‐1β [IL‐1β], interleukin‐6 [IL‐6], and interleukin‐17 [IL‐17]) have been proposed as modulators for key psychobiological substrates (such as hypothalamic–pituitary–adrenal axis dysregulation and monoamine neurotransmitter metabolism), which are implicated in the pathogenesis of anxiety and depression.^10^, ^11^, ^12^ Prior evidence suggests that inflammatory cytokines (such as IL‐1β, IL‐6, and TNF‐α) are related to anxiety and depression in cancers, including colorectal cancer, breast cancer, and pancreatic cancer.^13^, ^14^, ^15^, ^16^, ^17^ For instance, in colorectal cancer patients, serum IL‐1β, IL‐6, interleukin‐8 (IL‐8), and TNF‐α are positively correlated with increased anxiety and depression.^14^ In pancreatic cancer patients, patients with depression exhibit higher serum IL‐6 levels than patients without depression.^16^ As for NSCLC patients, only one previous study unravels that TNF‐α is higher in stage IV NSCLC patients with major depressive disorder than those without major depressive disorder.^18^ However, this previous study focuses on advanced NSCLC patients with major depressive disorders; furthermore, its sample size is relatively small (*N* = 55). Therefore, it is necessary to explore the associations of inflammatory cytokines with anxiety and depression in NSCLC survivors, while no relevant studies are available yet.

In the current study, we enrolled 217 NSCLC survivors to evaluate levels of TNF‐α, IL‐1β, IL‐6, and IL‐17 and their correlations with anxiety and depression.

## 2 MATERIALS AND METHODS

### 2.1 Participants

Between January 2019 and December 2019, 217 NSCLC survivors who came to our hospital for return visit were consecutively recruited in this study. The eligibility criteria included (1) diagnosed as primary NSCLC; (2) previously underwent surgical resection with curative intent (TNM stages I–III); (3) age within 18–80 years; (4) patient was alive without evidence of disease relapse at the time of recruitment; and (5) able to complete study assessment of anxiety and depression. Patients were excluded if they had any of the following conditions: (1) history of serious neurological disease; (2) history of serious mental illness or cognitive impairment; (3) complicated with other cancers; (4) known immune system disease, active infection, or inflammatory diseases; and (5) pregnant or lactating female patients. Besides, 200 controls were screened from the subjects who underwent healthy examination in our hospital from October 2019 to December 2019. The screening criteria for controls were (1) aged 50–80 and had no history of cancers; (2) no history of neurological disease, serious mental illness, or cognitive impairment; (3) no immune system disease, active infection, or inflammatory diseases; and (4) non‐pregnant or non‐lactating women. The controls were age‐ and gender‐matched with the recruited NSCLC survivors, which was achieved by controlling a sex ratio of 3:1 (male/female) and limiting controls' age to 50–80 years. The present study was approved by the Institutional Review Board of our hospital, and all participants provided the written informed consents.

### 2.2 Data collection

Demographic information and medical history of NSCLC survivors and controls were collected by their self‐reporting, which included age, gender, marriage status, employment status, level of education, smoking history, drinking history, and comorbidities (hypertension, hyperlipidemia, and diabetes). Duration after surgery and tumor features at surgery of NSCLC survivors (including pathological differentiation, tumor size, lymph node [LYN] metastasis, and TNM stage) were collected by medical chart review.

### 2.3 Blood sample collection

Peripheral blood samples of NSCLC survivors and controls were collected after they signed the informed consents. After that, the blood samples were allowed to clot by leaving it undisturbed at room temperature (usually 15–30 min), followed by centrifuging at 2,000×*g* for 10 min in a refrigerated centrifuge. Afterwards, serum samples were obtained from the resulting supernatant, and then, they were immediately transferred into a clean polypropylene tube using a Pasteur pipette.

### 2.4 Enzyme‐linked immunosorbent assay

Subsequently, the inflammatory cytokines in serum, including TNF‐α, IL‐1β, IL‐6, and IL‐17, were determined by enzyme‐linked immunosorbent assay (ELISA) kits (Invitrogen, Carlsbad, California, USA). All ELISA procedures were performed according to the kits instructions. Briefly, the samples and standards were added to the 96‐well plate. Then, the plate was incubated with the immobilized antibody, and a sandwich is formed by adding the second (detector) antibody. After that, tetramethylbenzidine substrate solution was added in the plate to produce measurable signals. Lastly, stop solution was added in the plate to terminate the reaction, and the optical densities were read at absorbance of 450 nm using a Multiskan™ FC microplate reader (Thermo Fisher Scientific, Waltham, Massachusetts, USA).

### 2.5 Anxiety, depression assessment, and treatment

After enrollment, the anxiety and depression of participants were assessed using the Hospital Anxiety and Depression Scale (HADS).^19^, ^20^, ^21^ After given description of scale filling by the investigator, participants were required to complete the HADS by themselves. Then, researchers assessed the anxiety and depression of participants using HADS. The HADS consisted of two subscales: HADS for anxiety (HADS‐A) and HADS for depression (HADS‐D). There were 14 items in HADS, with seven items in each subscale. Each item was scored from 0 to 3, resulting total score ranging from 0 to 21 for anxiety and 0 to 21 for depression, respectively. The anxiety was defined as HADS‐A score ≥8, and the anxiety severity was classified as 0–7, no anxiety; 8–10, mild anxiety; 11–14, moderate anxiety; and 15–21, severe anxiety; the depression was defined as HADS‐D score ≥8, and the depression severity was classified as 0–7, no depression; 8–10, mild depression; 11–14, moderate depression; and 15–21, severe depression.^22^ NSCLC survivors with severe anxiety or depression received anti‐anxiety treatment or anti‐depressant treatment, respectively. For NSCLC survivors with moderate anxiety or depression, they received anti‐anxiety or anti‐depressant treatment according to their clinical status. Besides, if clinically indicated, NSCLC survivors received appropriate adjuvant therapy (such as chemotherapy and radiation therapy) based on the NCCN guideline of NSCLC.^23^

### 2.6 Statistical analysis

Continuous variables were described as mean with standard deviation (SD) or median with interquartile range (IQR), according to the data distribution checked by Kolmogorov–Smirnov(K) test. Categorical variables were described as number and percentage. Comparison of continuous variables between two groups was determined by Student's *t* test or Wilcoxon rank sum test. Comparison of categorical variables between two groups was determined by Chi‐square test or Wilcoxon rank sum test. Correlation between two continuous variables was determined by Spearman's rank correlation test. Multivariate logistic regression analysis was performed on factors related to anxiety and depression, and the forward stepwise (conditional) method was used to screen the independent factors in multivariate logistic regression analysis. *P* value <0.05 was considered as statistically significant.

## 3 RESULTS

### 3.1 Clinical features of controls and NSCLC survivors

The majority of clinical features were of no difference between NSCLC survivors and controls, including age (*P* = 0.291), gender (*P* = 0.935), marriage status (*P* = 0.426), employment status (*P* = 0.060), education level (*P* = 0.844), drinking history (*P* = 0.265), hypertension (*P* = 0.182), hyperlipidemia (*P* = 0.200), or diabetes (*P* = 0.645) (Table 1). Among them, the percentage of patients with smoking history (*P* = 0.001) was higher in NSCLC survivors compared with controls. Additionally, in NSCLC survivors, 49 (22.6%), 115 (53.0%), and 53 (24.4%) of them had well, moderate, and poor pathological differentiation, respectively; their median (IQR) tumor size was 5.0 (4.0–7.0) cm; 67 (30.9%) patients were of LYN metastasis; 62 (28.6%), 84 (38.7%), and 71 (32.7%) patients were at TNM stages I–III, respectively; the median (IQR) duration after surgery was 1.8 (0.9–2.6) years. Information about other detailed characteristics was exhibited in Table 1.

**TABLE 1:** Characteristics of NSCLC survivors and controls

Items	Controls (*N* = 200)	NSCLC survivors (*N* = 217)	*P* value
Age (years), mean±SD	62.9 ± 5.7	63.8 ± 9.5	0.291
Gender, No. (%)			0.935
Female	50 (25.0)	55 (25.3)	
Male	150 (75.0)	162 (74.7)	
Marry status, No. (%)			0.426
Single/divorced/widowed	60 (30.0)	73 (33.6)	
Married	140 (70.0)	144 (66.4)	
Employment status, No. (%)			0.060
Unemployed	151 (75.5)	180 (82.9)	
Employed	49 (24.5)	37 (17.1)	
Level of education, No. (%)			0.844
Primary school or less	23 (11.5)	25 (11.5)	
High school	95 (47.5)	106 (48.8)	
Undergraduate	62 (31.0)	64 (29.5)	
Graduate or above	20 (10.0)	22 (10.2)	
History of smoke, No. (%)	73 (36.5)	114 (52.5)	0.001
History of drink, No. (%)	75 (37.6)	93 (42.9)	0.265
Hypertension, No. (%)	64 (32.0)	83 (38.2)	0.182
Hyperlipidemia, No. (%)	41 (20.5)	56 (25.8)	0.200
Diabetes, No. (%)	29 (14.5)	35 (16.1)	0.645
Pathological differentiation, No. (%)			
Well	‐	49 (22.6)	‐
Moderate	‐	115 (53.0)	‐
Poor	‐	53 (24.4)	‐
Tumor size (cm), median (IQR)	‐	5.0 (4.0–7.0)	‐
LYN metastasis, No. (%)	‐	67 (30.9)	‐
TNM stage, No. (%)	‐		‐
I	‐	62 (28.6)	‐
II	‐	84 (38.7)	‐
III	‐	71 (32.7)	‐
Duration after surgery (years), median (IQR)	‐	1.8 (0.9–2.6)	‐

Abbreviations: IQR, interquartile range; LYN, lymph node; NSCLC, non‐small cell lung cancer; SD, standard deviation.

### 3.2 Anxiety and depression between controls and NSCLC survivors

**FIGURE 1:**
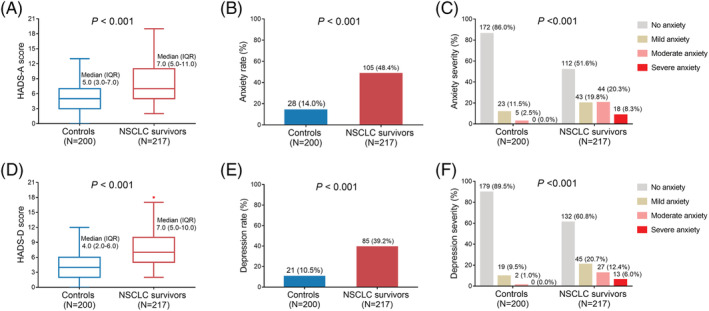
Difference in anxiety and depression between controls and NSCLC survivors. Comparisons of HADS‐A score (A), anxiety rate (B), anxiety severity (C), HADS‐D score (D), depression rate (E), and depression severity (F) between controls and NSCLC survivors. HADS‐A, Hospital Anxiety and Depression Scale for anxiety; HADS‐D, Hospital Anxiety and Depression Scale for depressionNSCLC, non‐small cell lung cancer

HADS‐A score (median [IQR]: 7.0 [5.0–11.0] vs. 5.0 [3.0–7.0], *P* < 0.001) (Figure 1A), anxiety rate (48.4% vs. 14.0%, *P* < 0.001) (Figure 1B), and anxiety severity (*P* < 0.001) (Figure 1C) were all increased in NSCLC survivors than in controls. Regarding depression, HADS‐D score (median [IQR]: 7.0 [5.0–10.0] vs. 4.0 [2.0–6.0], *P* < 0.001) (Figure 1D), depression rate (39.2% vs. 10.5%, *P* < 0.001) (Figure 1E), and depression severity in NSCLC survivors (*P* < 0.001) (Figure 1F) were also elevated than that in controls.

### 3.3 Inflammatory cytokines between controls and NSCLC survivors

**FIGURE 2:**
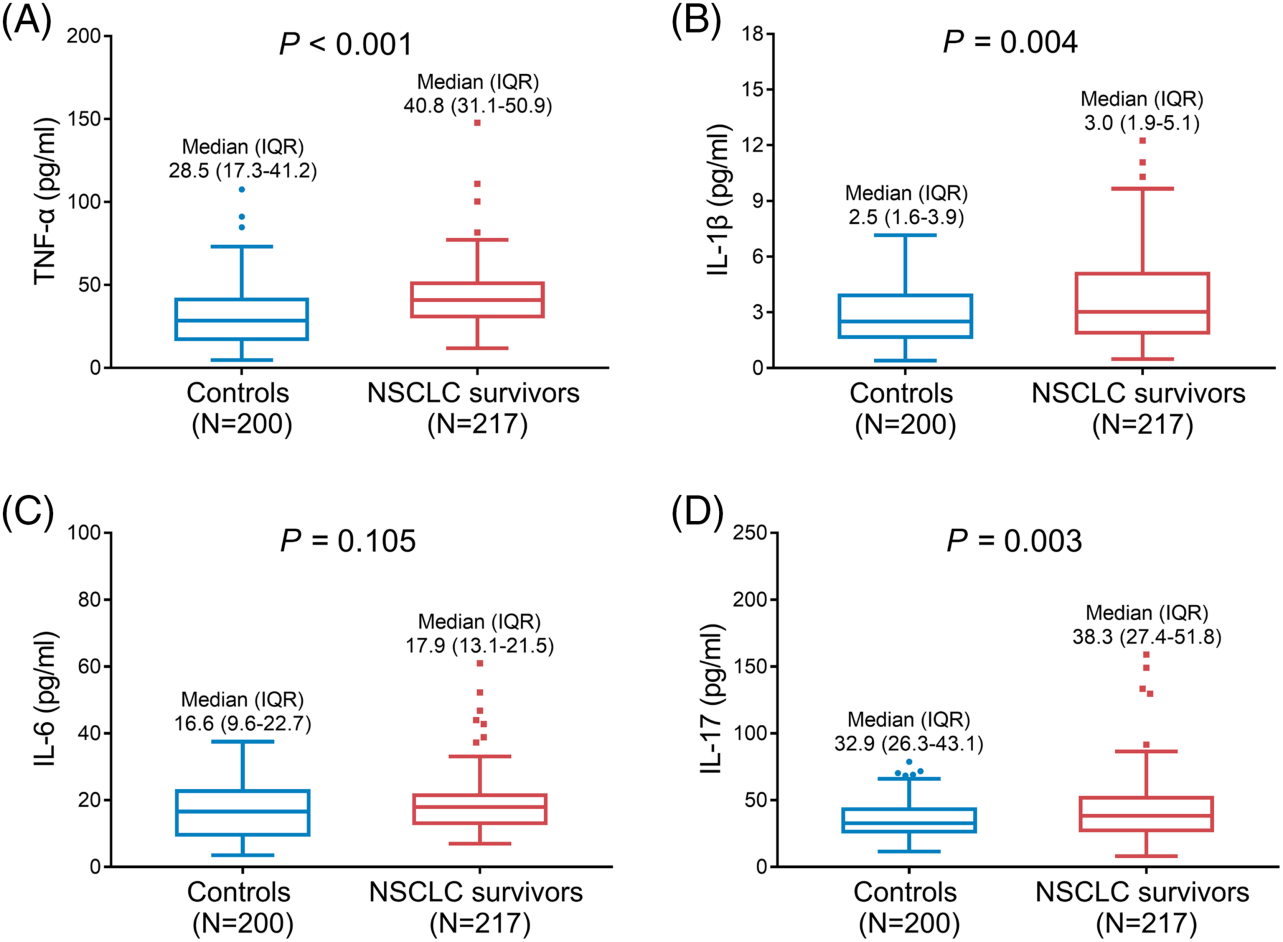
Difference in TNF‐α, IL‐1β, IL‐6, and IL‐17 between controls and NSCLC survivors. Comparisons of serum TNF‐α (A), IL‐1β (B), IL‐6 (C), and IL‐17 (D) between controls and NSCLC survivors. IL‐1β, interleukin‐1β; IL‐6, interleukin‐6; IL‐17, interleukin‐17; NSCLC, non‐small cell lung cancer; TNF‐α, tumor necrosis factor‐α

TNF‐α (median [IQR]: 40.8 [31.1–50.9] pg/ml vs. 28.5 [17.3–41.2] pg/ml, *P* < 0.001) (Figure 2A), IL‐1β (median [IQR]: 3.0 [1.9–5.1] pg/ml vs. 2.5 [1.6–3.9] pg/ml, *P* = 0.004) (Figure 2B), and IL‐17 (median [IQR]: 38.3 [27.4–51.8] pg/ml vs. 32.9 [26.3–43.1] pg/ml, *P* = 0.003) (Figure 2D) levels were all higher in NSCLC survivors than those in controls, while IL‐6 level (median [IQR]: 17.9 [13.1–21.5] pg/ml vs. 16.6 [9.6–22.7] pg/ml, *P* = 0.105) (Figure 2C) was similar between them.

### 3.4 Correlation of inflammatory cytokines with anxiety in NSCLC survivors

**FIGURE 3:**
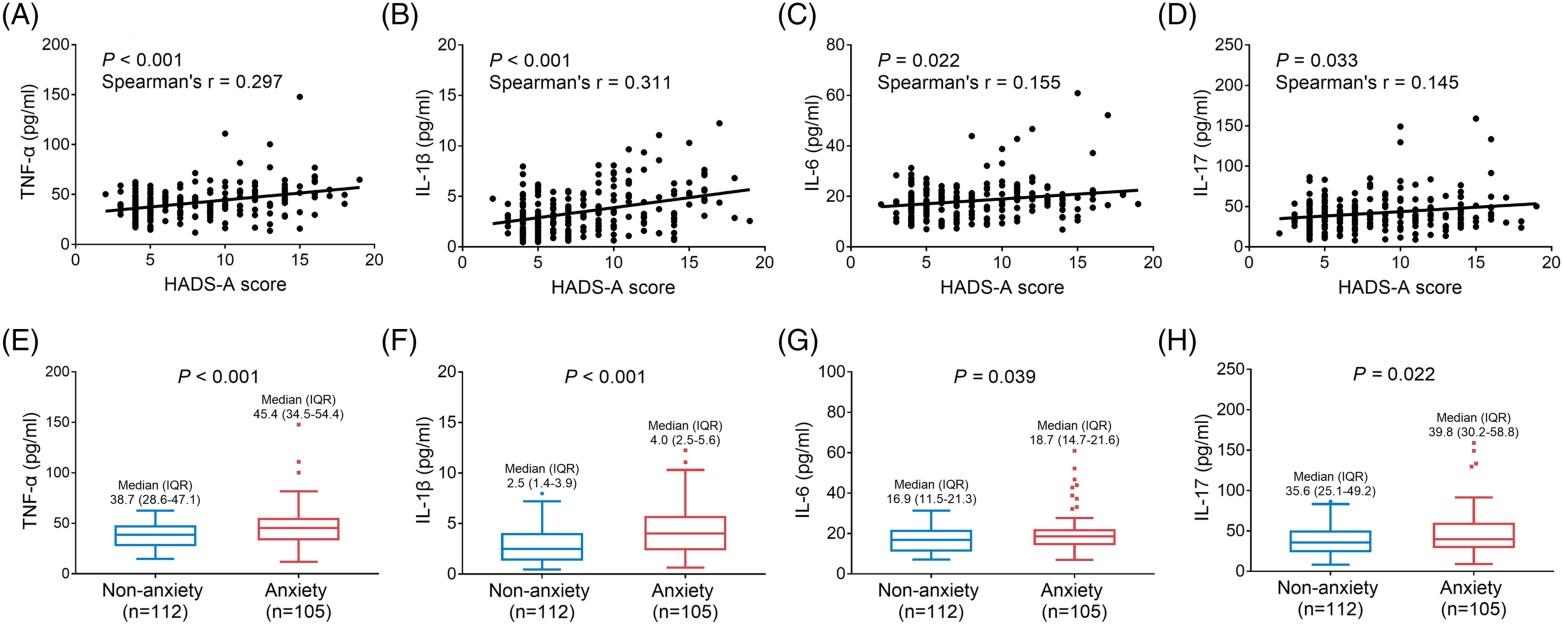
Correlations of TNF‐α, IL‐1β, IL‐6, and IL‐17 with anxiety in NSCLC survivors. Correlations of serum TNF‐α (A), IL‐1β (B), IL‐6 (C), and IL‐17 (D) levels with HADS‐A score. Comparisons of serum TNF‐α (E), IL‐1β (F), IL‐6 (G), and IL‐17 (H) levels between anxiety NSCLC survivors and non‐anxiety NSCLC survivors. IL‐1β, interleukin‐1β; IL‐6, interleukin‐6; IL‐17, interleukin‐17; NSCLC, non‐small cell lung cancer; TNF‐α, tumor necrosis factor‐α

TNF‐α (*P* < 0.001, *r* = 0.297) (Figure 3A), IL‐1β (*P* < 0.001, *r* = 0.311) (Figure 3B), IL‐6 (*P* = 0.022, *r* = 0.155) (Figure 3C), and IL‐17 (*P* = 0.033, *r* = 0.145) (Figure 3D) were all positively correlated with HADS‐A score; however, the correlation‐coefficients were weak. Meanwhile, TNF‐α (median [IQR]: 45.4 [34.5–54.4] pg/ml vs. 38.7 [28.6–47.1] pg/ml, *P* < 0.001) (Figure 3E), IL‐1β (median [IQR]: 4.0 [2.5–5.6] pg/ml vs. 2.5 [1.4–3.9] pg/ml, *P* < 0.001) (Figure 3F), IL‐6 (median [IQR]: 18.7 [14.7–21.6] pg/ml vs. 16.9 [11.5–21.3] pg/ml, *P* = 0.039) (Figure 3G), and IL‐17 (median [IQR]: 39.8 [30.2–58.8] pg/ml vs. 35.6 [25.1–49.2] pg/ml, *P* = 0.022) (Figure 3H) were all increased in anxiety NSCLC survivors compared to non‐anxiety NSCLC survivors. Notably, the correlations of TNF‐α and IL‐1β with anxiety seemed to be stronger than IL‐6 and IL‐17.

### 3.5 Independent factors correlated with anxiety in NSCLC survivors

Multivariate logistic regression analysis revealed that TNF‐α (odds ratio [OR] (95% confidence interval [CI]) = 1.031 [1.007–1.056], *P* = 0.012), IL‐1β (OR [95% CI] = 1.376 [1.159–1.633], *P* < 0.001), marriage (single/divorced/widowed) status (OR [95% CI] = 2.086 [1.064–4.090], *P* = 0.032), diabetes (OR [95% CI] = 2.948 [1.181–7.360], *P* = 0.021), higher TNM stage (OR [95% CI] = 2.047 [1.344–3.117], *P* = 0.001), and longer duration after surgery (OR [95% CI] = 1.414 [1.080–1.852], *P* = 0.012) were independently correlated with elevated anxiety risk in NSCLC survivors (Table 2).

**TABLE 2:** Independent anxiety‐related factors in NSCLC survivors

Items	Multivariate logistic regression (forward stepwise [conditional])	
	*P* value	OR (95%CI)
TNF‐α	0.012	1.031 (1.007–1.056)
IL‐1β	<0.001	1.376 (1.159–1.633)
Marry status (single/divorced/widowed)	0.032	2.086 (1.064–4.090)
Diabetes	0.021	2.948 (1.181–7.360)
Higher TNM stage	0.001	2.047 (1.344–3.117)
Longer duration after surgery	0.012	1.414 (1.080–1.852)

Abbreviations: CI, confidence interval; IL, interleukin; LYN, lymph node; NSCLC, non‐small cell lung cancer; OR, odds ratio; TNF, tumor necrosis factor.

### 3.6 Correlation of inflammatory cytokines with depression in NSCLC survivors

**FIGURE 4:**
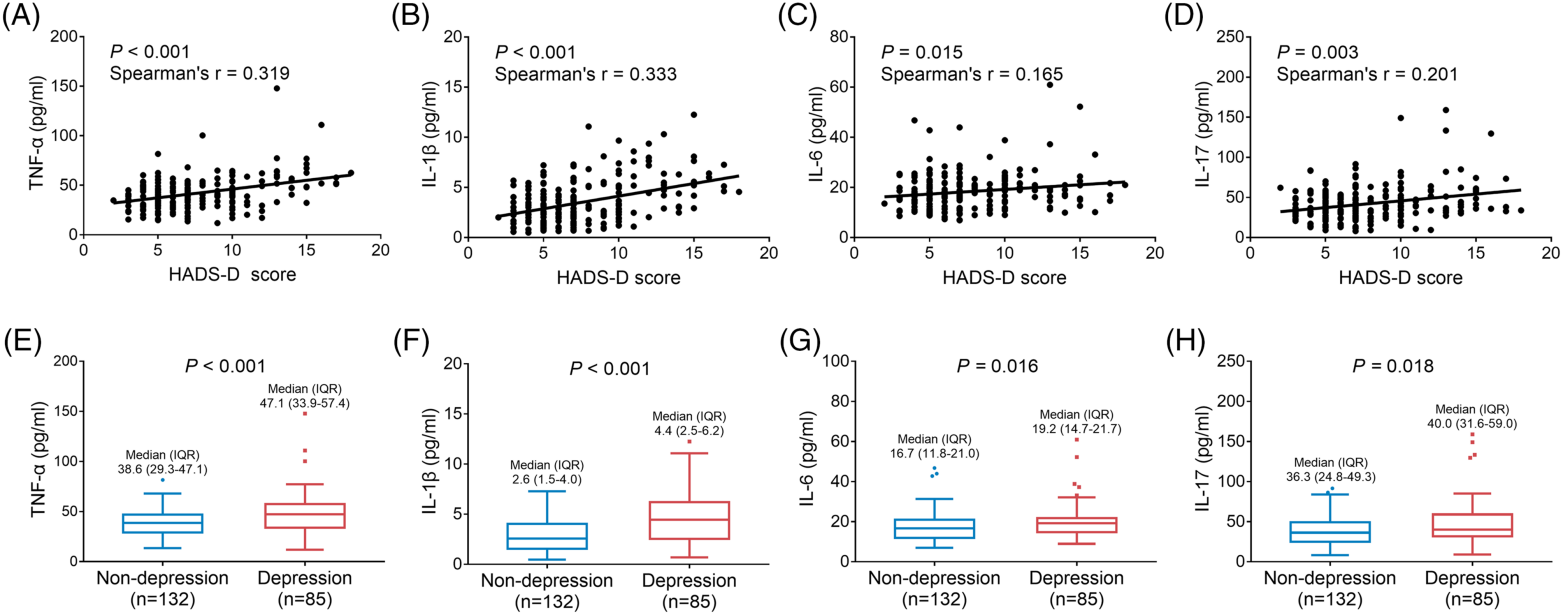
Correlations of TNF‐α, IL‐1β, IL‐6, and IL‐17 with depression in NSCLC survivors. Correlations of serum TNF‐α (A), IL‐1β (B), IL‐6 (C), and IL‐17 (D) levels with HADS‐D score. Comparisons of serum TNF‐α (E), IL‐1β (F), IL‐6 (G), and IL‐17 (H) levels between depression NSCLC survivors and non‐depression NSCLC survivors. IL‐1β, interleukin‐1β; IL‐6, interleukin‐6; IL‐17, interleukin‐17; NSCLC, non‐small cell lung cancer; TNF‐α, tumor necrosis factor‐α

TNF‐α (*P* < 0.001, *r* = 0.319) (Figure 4A), IL‐1β (*P* < 0.001, *r* = 0.333) (Figure 4B), IL‐6 (*P* = 0.015, *r* = 0.165) (Figure 4C), and IL‐17 (*P* = 0.003, *r* = 0.201) (Figure 4D) were positively correlated with HADS‐D score, but the correlation‐coefficients were weak. Meanwhile, TNF‐α (median [IQR]: 47.1 [33.9–57.4] pg/ml vs. 38.6 [29.3–47.1] pg/ml, *P* < 0.001) (Figure 4E), IL‐1β (median [IQR]: 4.4 [2.5–6.2] pg/ml vs. 2.6 [1.5–4.0] pg/ml, *P* < 0.001) (Figure 4F), IL‐6 (median [IQR]: 19.2 [14.7–21.7] pg/ml vs. 16.7 [11.8–21.0] pg/ml, *P* = 0.016) (Figure 4G), and IL‐17 (median [IQR]: 40.0 [31.6–59.0] pg/ml vs. 36.3 [24.8–49.3] pg/ml, *P* = 0.018) (Figure 4H) were elevated in depression NSCLC survivors compared to non‐depression NSCLC survivors. Of note, the correlations of TNF‐α and IL‐1β with depression were more obvious compared with IL‐6 and IL‐17.

### 3.7 Independent factors correlated with depression in NSCLC survivors

Multivariate logistic regression analysis disclosed that TNF‐α (OR [95% CI] = 1.035 [1.012–1.060], *P* = 0.003), IL‐1β (OR [95% CI] = 1.373 [1.165–1.618], *P* < 0.001), diabetes (OR [95% CI] = 2.713 [1.122–6.560], *P* = 0.027), higher TNM stage (OR [95% CI] = 1.637 [1.072–2.500], *P* = 0.022), and prolonged duration after surgery (OR [95% CI] = 1.355 [1.035–1.773], *P* = 0.027) were independently correlated with increased depression risk, while higher education level (OR [95% CI] = 0.662 [0.440–0.998], *P* = 0.049) was independently correlated with decreased depression risk in NSCLC survivors (Table 3).

**TABLE 3:** Independent depression‐related factors in NSCLC survivors

Items	Multivariate logistic regression (forward stepwise [conditional])	
	*P* value	OR (95%CI)
TNF‐α	0.003	1.035 (1.012–1.060)
IL‐1β	<0.001	1.373 (1.165–1.618)
Higher level of education	0.049	0.662 (0.440–0.998)
Diabetes	0.027	2.713 (1.122–6.560)
Higher TNM stage	0.022	1.637 (1.072–2.500)
Longer duration after surgery	0.027	1.355 (1.035–1.773)

Abbreviations: CI, confidence interval; IL, interleukin; LYN, lymph node; NSCLC, non‐small cell lung cancer; OR, odds ratio; TNF, tumor necrosis factor.

### 3.8 Subgroup analyses

In order to clarify the influence of adjuvant chemotherapy and TNM stage on the correlation between inflammatory cytokines and anxiety/depression, subgroup analyses were conducted. Most of the inflammatory cytokines were correlated with HADS‐A and HADS‐D scores in patients with adjuvant chemotherapy (*n* = 159), but not in patients without adjuvant chemotherapy (*n* = 58) (Table 4). Besides, most of the inflammatory cytokines were correlated with HADS‐A and/or HADS‐D scores in patients with TNM stage II (*n* = 84), and in patients with TNM stage III (*n* = 71), while not in patients with TNM stage I (*n* = 62).

**TABLE 4:** Subgroup analyses

Items		TNF‐α	IL‐1β	IL‐6	IL‐17
Patients with adjuvant chemotherapy (*n* = 159)					
HADS‐A score	*r* coefficient	0.325	0.297	0.157	0.071
	*P* value	<0.001	<0.001	0.048	0.375
HADS‐D score	*r* coefficient	0.360	0.352	0.152	0.179
	*P* value	<0.001	<0.001	0.055	0.024
Patients without adjuvant chemotherapy (*n* = 58)					
HADS‐A score	*r* coefficient	0.269	0.238	0.116	0.212
	*P* value	0.041	0.073	0.385	0.111
HADS‐D score	*r* coefficient	0.239	0.181	0.153	0.151
	*P* value	0.070	0.174	0.250	0.257
Patients with TNM stage I (*n* = 62)					
HADS‐A score	*r* coefficient	0.241	0.199	0.017	0.188
	*P* value	0.059	0.121	0.896	0.144
HADS‐D score	*r* coefficient	0.272	0.247	0.105	0.173
	*P* value	0.032	0.053	0.416	0.179
Patients with TNM stage II (*n* = 84)					
HADS‐A score	*r* coefficient	0.197	0.331	0.172	0.099
	*P* value	0.073	0.002	0.117	0.368
HADS‐D score	*r* coefficient	0.286	0.307	0.233	0.158
	*P* value	0.008	0.004	0.033	0.151
Patients with TNM stage III (n = 71)					
HADS‐A score	*r* coefficient	0.431	0.280	0.235	0.064
	*P* value	<0.001	0.018	0.048	0.595
HADS‐D score	*r* coefficient	0.397	0.362	0.106	0.214
	*P* value	0.001	0.002	0.378	0.074

Abbreviations: HADS‐A, Hospital Anxiety and Depression Scale‐anxiety; HADS‐D, Hospital Anxiety and Depression Scale‐depression; IL, interleukin; TNF, tumor necrosis factor.

## 4 DISCUSSION

Anxiety and depression are common cancer‐related psychological complications in lung cancer patients.^6^, ^7^, ^8^ In line with previous studies, the current study disclosed that anxiety and depression were more prevalent and severe in NSCLC survivors compared with controls. This finding might be explained by several reasons: (1) diagnosis of the life‐threatening NSCLC, physical deterioration, and interfered family/social life probably evoked a sense of hopelessness, fear, social isolation, loss, or mental discomfort in NSCLC survivors, which predisposed to the development of anxiety and depression.^5^, ^24^ (2) Along with disease progression and aggressive treatments (surgical resection and chemoradiotherapy), NSCLC survivors suffered from severe symptoms of NSCLC (such as dyspnea, anorexia, chest pain, and fatigue), declined daily activities after surgical resection, chemoradiotherapy‐related cytotoxicity, and financial burden, which resulted in the development of anxiety and depression in NSCLC survivors.^5^, ^24^

Inflammatory cytokines (especially TNF‐α, IL‐1β, IL‐6, and IL‐17) in the brain modulate its downstream inflammatory signaling pathway including NF‐κB, which disturb monoamine metabolism, elevate excitotoxicity, and reduce the release of tropic factors, exerting a profound impact on psychological state, including anxiety and depression in cancer patients.^7^, ^8^ In the clinical aspects, preceding evidence suggests the association of inflammatory cytokines (such as TNF‐α, IL‐8, and IL‐6) with anxiety and depression in cancers, such as colorectal cancer, breast cancer, and pancreatic cancer.^13^, ^14^, ^15^, ^16^, ^17^ For example, serum TNF‐α, IL‐1β, IL‐6, and IL‐8 are positively associated with elevated HADS score in colorectal cancer patients.^15^ Another study illuminates that breast cancer patients with aggravating depressive symptoms exhibit higher serum level of TNF‐α and IL‐6.^17^ As for NSCLC patients, only one previous study reveals that serum TNF‐α is positively associated with major depressive disorder.^18^ This study only enrolls advanced NSCLC patients with major depressive disorder, which has relatively small sample size (*N* = 55). The above‐mentioned information shows that relevant studies are still lacking regarding the association of inflammatory cytokines with anxiety and depression in NSCLC survivors. In the current study, we detected and compared the serum inflammatory cytokines in 217 NSCLC survivors and 200 controls. The results displayed that serum TNF‐α, IL‐1β, and IL‐17 levels were increased, while serum IL‐6 level was of no difference in NSCLC survivors compared with controls. The following were possible explanations: (1) Dysregulated immune host cells (such as infiltrated inflammatory cells and endothelial cells) after surgery produced high levels of proinflammatory cytokines in the tumor microenvironment; thereby, NSCLC survivors had higher serum TNF‐α, IL‐1β, and IL‐17 levels than controls.^25^ (2) Tissue destruction by surgical resection might induce the release of damage‐associated molecular patterns in response to treatment‐associated cell death, which activated the expression of the transcription factor nuclear factor‐κ‐β and exaggerated the production of proinflammatory cytokines, thereby contributing to increased serum TNF‐α, IL‐1β, and IL‐17 levels in NSCLC survivors compared with controls.^26^ (3) As the predominant role of IL‐6 was to sustain the balance of immunity and inflammation, the dysregulation of IL‐6 level was rare in lung cancer patients, while this hypothetical explanation needed further validation.

A few studies also reveal the linkage of inflammatory cytokines with anxiety and/or depression in lung cancer patients.^18^, ^27^, ^28^ The current study also revealed that serum TNF‐α, IL‐1β, IL‐6, and IL‐17 were positively correlated with anxiety and depression risks in NSCLC survivors. Possible explanations could be as follows: (1) Upregulated levels of proinflammatory cytokines might trigger the expression of the serotonin transporter, elevate the reuptake of serotonin, and reduce extracellular synaptic serotonin concentrations and serotonin neurotransmission, which contributed to anxiety and depression in NSCLC survivors; thus, serum TNF‐α, IL‐1β, IL‐6, and IL‐17 were positively correlated with anxiety and depression in NSCLC survivors.^10^, ^29^ (2) Proinflammatory cytokines might facilitate the dysregulation of hypothalamic–pituitary–adrenal axis, which stimulated the secretion of corticotrophin‐releasing hormone, adrenocorticotropic hormone and cortisol that impacted neuroendocrine, neural, and immune pathways associated with anxiety and depression. Hence, serum TNF‐α, IL‐1β, IL‐6, and IL‐17 were positively related to anxiety and depression in NSCLC survivors.^29^, ^30^ Of note, serum TNF‐α and IL‐1β were more strongly positively associated with anxiety and depression than serum IL‐6 and IL‐17 in NSCLC survivors, which might be explained by that IL‐6 and IL‐17 might be indirectly involved in the pathophysiology of anxiety and depression by modulating other inflammatory pathways and mediating the release of other cytokines.^11^

In addition, the multivariate logistic regression analyses of independent anxiety‐related and depression‐related factors in NSCLC survivors were performed to adjust covariates. The results indicated that TNF‐α and IL‐1β were independently correlated with increased anxiety and depression risks in NSCLC survivors, which could be explained by that as observed in our study, the correlations of TNF‐α and IL‐1β with anxiety and depression tended to be more evident than IL‐6 and IL‐17; meanwhile, IL‐6 and IL‐7 were highly correlated with TNF‐α and IL‐1β. Therefore, after adjusting covariates by multivariate logistic regression analyses, only TNF‐α and IL‐1β were independently correlated with increased anxiety and depression risks in NSCLC survivors.

Interestingly, we also observed that most of the inflammatory cytokines were correlated with HADS‐A and HADS‐D scores in patients with adjuvant chemotherapy (*n* = 159), but not in patients without adjuvant chemotherapy (*n* = 58). The possible explanations were (1) patients with adjuvant chemotherapy reflected a higher severe disease condition that enlarged the dysregulation of inflammatory cytokines and HADS‐A/D score, at the same time amplifying their correlation and (2) the small sample size of patients without adjuvant chemotherapy (*n* = 58) reduced statistical power. Besides, most of the inflammatory cytokines were correlated with HADS‐A and HADS‐D scores in patients with TNM stage II (*n* = 84) and stage III (*n* = 71), while not in patients with TNM stage I (*n* = 62). The reason might be that higher TNM stage enlarged the dysregulation of inflammatory cytokines and HADS‐A/D score, further amplifying their correlation. Moreover, according to previous studies, inflammatory cytokines could cause anxiety and depression via regulating hormones, neurotransmitters, and so on. Meanwhile, other studies also observe that anxiety would increase inflammatory cytokines. Therefore, it is not correct to conclude that dysregulation of inflammatory cytokines is the result or cause of anxiety. It is possible they are interacted and getting worse during the disease course of NSCLC. Lastly, we hypothesized that inflammatory cytokine is a trigger of anxiety/depression, but not the only one. Other factors such as concerning of disease relapse, family burden are all potential causes of anxiety/depression. Therefore, longer duration of NSCLC increases patient's concerns about disease relapse and accumulating financial burden; thus, it was related to higher anxiety and depression.

Nonetheless, some limitations should be noted in the current study. First, since all the participates were recruited from a single center, a sampling bias was present in this data. Second, anxiety and depression of participants were assessed using a self‐administered questionnaire (HADS), which might result in a response bias; therefore, more assessment tools for anxiety and depression were needed for validating our findings. Third, some confounding factors (such as adjuvant therapy and anti‐anxiety/anti‐depression therapy if clinically indicated) were not included in the analyses. Fourth, as it was difficult to recruit controls, the sample size was relatively small, which might decrease the statistical power. Fifth, to eliminate the strong confounding effect of disease relapse on anxiety and depression, NSCLC patients with evidence of disease relapse were excluded at the time of recruitment; thereby, our findings might be not applicable to these patients.

To conclude, serum TNF‐α, IL‐1β, and IL‐17 levels are highly expressed, which are closely related to elevated anxiety and depression risks to some extent in NSCLC survivors. These indicated the potential involvement of inflammatory cytokines in the development of anxiety and depression in these patients, which might assist the screening of anxiety/depression, the management of mental health, and the improvement of prognosis in the clinical setting.

## CONFLICT OF INTEREST

The authors declared that they have no conflict of interest with the contents of this article.

## AUTHOR CONTRIBUTIONS

Meifang Liu designed the study and performed the research; Xuesong Liu and Yao Li collected and analyzed the data; Meifang Liu, Xuesong Liu, and Yao Li wrote and reviewed the paper.

## ETHICS STATEMENT

The present study was approved by the Institutional Review Board of our hospital, and all participants provided the written informed consents.

## Data Availability

The data that support the findings of this study are available from the corresponding author upon reasonable request.
